# Does authoritarianism necessarily stifle creativity? The role of discipline-focused authoritarian leadership

**DOI:** 10.3389/fpsyg.2022.1037102

**Published:** 2022-10-26

**Authors:** Honglei Zhao, Qingming Su, Ming Lou, Chuqi Hang, Li Zhang

**Affiliations:** ^1^School of Management, Harbin Institute of Technology, Harbin, China; ^2^Heilongjiang Provincial Government Offices Administration, Harbin, China

**Keywords:** authoritarian leadership, creative self-efficiency, appointment event criticality, event system theory, employee creativity

## Abstract

A burgeoning body of research has shown that authoritarian leadership (AL) embodies the characteristics of “light” and “dark,” meaning that it does not always have a negative impact on employees’ creative activities. However, studies explaining this potential positive effect are insufficient. To extend the AL and creativity literature, we draw on self-determination theory and event system theory, and elicit discipline-focused AL and appointment event criticality to examine whether, when, and how authoritarian leaders affect employee creativity positively. With time-lagged data collected from 435 employees and their direct leaders in China, we found that discipline-focused AL has an indirect positive effect on employee creativity through creative self-efficacy. Additionally, appointment event criticality strengthens the positive relationship between discipline-focused AL and creative self-efficiency, and the indirect impact of discipline-focused AL on employee creativity through creative self-efficiency. The theoretical and practical implications are discussed.

## Introduction

Creativity is considered the engine of organizational success (Anderson et al., 2004, 2014), and is defined as a useful and innovative idea for a person or group of people to work together (Zhou and George, 2001). Among the many factors that affect employee creativity and innovation, a leader’s behavior and style often play a crucial role, especially as the employee’s immediate supervisor (Niu et al., 2018; Zhang et al., 2021). Numerous studies have explored the impact of positive leadership on employee creativity (Bai et al., 2016; Guo et al., 2018), such as transformational leadership and humble leadership, which have been widely proven to promote creativity (e.g., Shin and Zhou, 2003; Aasland et al., 2010). Despite scholars’ devotion and contribution, many studies on these types of leadership and creativity are duplicated and the conclusions are similar (Zhao et al., 2022). Recently, scholars have shifted attention to destructive leadership, among which authoritarian leadership (AL) is the most representative (Wang and Xing, 2019). However, in explaining the impact of AL on employee creativity, we raise two questions that require further explanation.

First, the influence of authoritarianism on employee creativity is still controversial (Guo et al., 2018). Specifically, the positive effects of AL on creativity under-explained. Most research views AL as the “dark” side of leadership (Thom, 2006; Aryee et al., 2007). Accordingly, many scholars believe that AL is harmful to subordinates’ creativity and have proposed a simple negative linear relationship between the two (e.g., Gu et al., 2018; Guo et al., 2018). However, it seems that AL often plays a positive role in Chinese organizations (Chen and Farh, 2010), which is reflected in the potential positive impact on employee creativity (Zhao et al., 2022). For example, Gu et al. (2020) found a positive effect of AL on employee creativity under the conditions of high-benevolent and high-morality leadership. Zhang et al. (2021) indicated that AL has a positive effect on employee innovation behavior within a certain range in the Chinese organizational culture. Zhao et al. (2022) also proposed a positive influence of AL on employee creativity through a qualitative study. One explanation is that AL, when treated as a dimension of paternalistic leadership, may be considered as an effort to help and guide the staff (Leung et al., 2014). Others argued that such controlling behavior can be considered a stress-induced agent that can trigger a stimulation (Aryee et al., 2007; Gu et al., 2020; Cheng et al., 2021), especially the employees’ sense of competence (Zhao et al., 2021, 2022) because individuals tend to overcome stress to satisfy their basic psychology needs (Cranmer et al., 2018).

We argue that AL needs to be viewed dialectically in the process of influencing creativity. It is worth noting that some scholars have identified two dimensions of AL according to the different control focus (e.g., Cheng and Chou, 2005; Chou et al., 2010; Chou and Cheng, 2014), namely discipline-focused AL and dominance-focused AL, which refer to strict discipline and dominant control, respectively (Cheng and Chou, 2005). Furthermore, these studies confirmed that discipline-focused AL has a positive effect on employees in terms of task performance and perceived rights (Cheng et al., 2006). Based on the above, we propose that discipline-focused AL may help explain why AL doesn’t necessarily stifle employee creativity and may even have a positive effect. This is the first objective of this research.

Second, previous research has taken a primarily static view of the relationship between leadership and employee creativity. They have focused more attention to internal stability characteristics of entities (individuals, teams, and organizations) (Liu and Liu, 2017), but not on the potential impact of changes in dynamic events, whose strength may change over time and be always present (Morgeson et al., 2015). Along this line, authoritarian control does not necessarily occur isolated from workplace events, which complicates the study of AL impact on creativity. Previous studies on creativity believe that employee creativity should be influenced by long-term and stable management, or by a relatively familiar and stable working environment. However, given the dynamic organizational environment today, where frequent workplace events disrupt the routine guiding employees’ behaviors (Chen et al., 2021), this ideal state of sustained stability and certainty is not permanent. Through a comprehensive review of previous studies considering context, it is obvious that there is a lack of empirical research on discrete events as context. The process of AL affecting employee creativity may vary according to specific, independent, and discrete events.

In our research, we quote the criticality of a new workplace event as our study’s boundary condition that is appointment event criticality. Appointment event refers to the experiences by an employee is designated for solving a new problem or undertaking a task as a team member (Zhao et al., 2022), and the criticality of which reflects “the importance, necessity, or priority of these events” (Morgeson et al., 2015; Liu et al., 2018). In the workplace, an employee may be appointed to take on a role at some point by his leader, and the experience may affect how the employee perceives his leaders. However, although most people constantly experience appointment events, this topic has not received enough attention. Therefore, our research aims to enrich the AL study on employee creativity by examining the effect of “appointment event criticality” as a moderator variable.

We develop a model according to the self-determination theory (SDT) and the event system theory (EST). Our basic argument is that discipline-forced AL could meet employees’ basic needs (i.e., competence and relatedness) and stimulate individual motivation, which positively influences employee creativity by promoting creative self-efficacy. Overall, our research makes three contributions to pertinent literature. First, our study enriches the current AL literature by uncovering a potentially “light” side of AL, which emphasizes different types of controlling behavior that arise from discipline-focused AL. Second, our study develops an understanding of leaders’ control behaviors and employee creativity. We argue that not all control behaviors are harmful to employee creativity. Requiring employees to set higher goals and adhere to organizational norms may be beneficial to employee creativity. Third, our research derives “appointment event criticality” as a situational variable from an integrative theory building perspective (Morgeson et al., 2015). This may enable the development of more fine-grained leadership and creativity theories, enhancing their explanatory power and impact.

## Theory and hypothesis development

### Discipline-focused authoritarian leadership

Authoritarian leadership indicates that the leader emphasizes absolute authority, who tightly controls subordinates and demands their unreserved obedience (Farh and Cheng, 2000). AL originates from the Chinese Confucian culture and Legalist culture and exists widely in the workplace, it was once thought to be the clearest and the most distinct leadership in Chinese enterprises. This leadership style emphasizes the authority of leaders and that employees obey orders unconditionally through strict control (Zheng et al., 2021). Most studies show that these characteristics of AL lead to negative behaviors and emotions of employees, but there is no consensus on the positive impact on employee behaviors and attitudes. The reason for this inconsistency may be that AL has the properties of both “light” and “dark” at the same time regarding job demands such as rigorousness, task monitoring, regulation and structuring, issue rules, control and dominance, and information manipulation. The vagueness of connotation has not been clarified (Farh and Cheng, 2000; Aryee et al., 2007; Farh et al., 2008; Chen and Farh, 2010).

To make the concept of AL clearer, Chou et al. (2010) proposed a two-factor solution, which divides the broad sense of controlling authoritarianism into “juan-chiuan” and “shang-yan,” namely, dominance-focused AL and discipline-focused AL (Chou and Cheng, 2014). The former is viewed as the “dark” side, highlighting tight control and social distance in a hierarchy where leaders assert their dominance and authority to meet their own needs. In contrast, the latter is considered the “bright” side, emphasizing disciplines and rules in an organization to achieve personal growth and collective goals that benefit employees and the organization (Cheng and Chou, 2005; Aryee et al., 2007; Chou et al., 2010; Chen, 2011). This study explains the “bright” side of AL and its positive impact on employee creativity, so we focus on the “discipline-focused AL.” This leadership style was referred to as the “stress challenge” in later studies (Cheng et al., 2021), representing the leader’s behavior that enable employees to overcome difficulties and achieve high standards, even when the odds are against them. The goal of discipline-focused AL is to benefit the collective from the leader’s authority and power (Chou and Cheng, 2014).

Consequently, we argue that “discipline-focused AL” brings up a new perspective for understanding the relationship between employee creativity and AL, and a theoretical explanation for previous inconsistent findings when examining the relationship between the two.

### Discipline-focused authoritarian leadership and creative self-efficacy

A pioneering study on the creative self-efficacy of employees was conducted by Tierney and Farmer (2002). They first made the concept of creative self-efficacy, which is defined as an individual’s evaluation of whether he or she has the ability and confidence to produce creative results when engaged in specific tasks and reflects the individual’s self-belief or self-expectation in creative activities. Creative self-efficacy is based on general self-efficacy and the creative theory, but is different from general self-efficacy, which reflects a person’s overall belief in cross-domain ability (Zhou et al., 2012). Creative self-efficacy emphasizes an employees’ belief in the realization of their creativity, including belief in creative approaches to work and belief in obtaining creative results, not just for the outcome of the action, but for the process of behavior (Zhang and Zhou, 2014). Conceptually, creative self-efficacy reveals two psychological states of employee creative contribution —— “I want” and “I can,” which is consistent with the intrinsic motivation and the satisfaction of basic psychological needs defined by the SDT (Deci and Ryan, 2000; Tierney and Farmer, 2002).

The extant literature has suggested that leadership, as a key contextual factor in organizations, is an important factor affecting individual self-evaluation and behavior (Li et al., 2015). In our study, discipline-focused authoritarian leader presents a more active behavioral pattern as contextual stimuli. On the one hand, strict management, high performance expectations, and high standards will stimulate employees’ sense of “competence,” inducing a belief that self-worth and self-fulfillment can be achieved through creative work performance. Discipline-focused authoritarian leaders tend to give their employees more complex and challenging tasks. Previous research shows that the complexity of a job reflects a person’s ability, and employees will evaluate their ability accordingly. The more complex or difficult a task an employee performs, the more likely he or she will have a higher assessment of his or her abilities (Gist and Mitchell, 1992). Moreover, in the process, employees can discover nuances and tricks, thus becoming more confident in completing tasks creatively (Karwowski et al., 2018).

On the other hand, discipline-focused AL generally aims to cultivate subordinates, improve their ability, and help them become excellent by guiding them (Wang and Xing, 2019; Cheng et al., 2021). When discipline-focused AL monitors subordinates’ work tasks, demands high performance, and maintains organizational norms, subordinates will show higher self-requirements and job engagement, and employees’ identification with the leader will be enhanced. In this process, the psychological distance between leaders and employees is “narrowed.” Subordinates see their leader as a “strict father.” As previous research has shown that attention and trust from organizations can make individuals happier (Joo et al., 2014), employees’ work enthusiasm is constantly stimulated due to of attention and trust from the leader. Employees’ high-identity recognization will promote a strong positive emotion toward the organization, thus improving intrinsic motivation and stimulating a strong desire to adopt a more flexible cognitive process when solving problems. The satisfied basic psychological needs of “relatedness” will prompt employees to pursue creative contributions.

Zhao et al. (2022) demonstrate that “competence” and “relatedness” are keys to understanding how AL influences employee creativity because they can easily trigger employees’ corresponding internal perceptions. In our study, we argue that creative self-efficacy is a positive internal perception. According to the SDT, discipline-focused AL satisfies employees’ needs for “competence” and “relatedness,” stimulating the intrinsic motivation of employees and making employees feel a strong sense of self-determination, which is helpful to improve their creative self-efficacy. Consequently, employees have enough confidence to deal with challenges at work and have the faith to complete tasks creatively. Therefore, we propose that employees subordinated to discipline-focused AL have a high level of creative self-efficacy. We hypothesize the following:

*Hypothesis 1: Discipline-focused AL has a positive effect on creative self-efficacy.*

### The mediating role of creative self-efficacy

According to social cognitive theory, individual cognition, behavior, and context factors affect each other (Bandura, 1989). Creative self-efficacy, an individual cognitive variable, has a profound impact on employee creativity and plays an important mediating role in the process of situational stimulation on behavior (Gong et al., 2009). Previous studies have confirmed that employees’ creative self-efficacy is positively related to their leader’s support behavior (Mittal and Dhar, 2015). An employee with strong creative self-efficacy is good at actively acquiring new knowledge at work. They are confident in their intelligence and ability to create, dare to try different things at work and have the courage to put forward and express new ideas (Han and Bai, 2020). Moreover, they are more tolerant of failures in attempts (Tierney and Farmer, 2002). The higher the level of creative self-efficacy, the more likely employees to persevere to achieve goals without flinching (Liu et al., 2016; Newman et al., 2018). The mediating role of creative self-efficacy in employee innovation has been widely recognized (Teng et al., 2020). In contrast, people whose creative self-efficacy is low lack the courage and determination to try new things and the confidence to produce creative results. Therefore, creative self-efficacy has a positive impact on employee creativity. A large number of empirical studies have shown that creativity self-efficacy as an internal drive of employee creativity has a positive impact on it (e.g., Haase et al., 2018; Newman et al., 2018; Bicer et al., 2020).

In our model, we theorize that discipline-focused AL as a contextual stimulus causes a cognitive state of employee manifesting in creative self-efficacy, where the employee’s two basic psychological needs for “relatedness” and “competency” are satisfied. Subsequently, creative self-efficacy should motivate employees to produce more creativity. We hypothesize the following:

*Hypothesis 2: creative self-efficacy mediates the relationship between discipline-focused AL and employee creativity.*

### Synergistic effect of appointment event criticality and discipline-focused authoritarian leadership

Event system theory suggests that it is necessary to understand how event characteristics combine with entities’ (e.g., individuals, teams, or organizations) internal features to influence employee perception and behavior (Morgeson et al., 2015; Jiang et al., 2019; Liu et al., 2021). This comprehensive method can further promote the development of organization theory and enhance its explanatory power and influence (Chen et al., 2021; Liu et al., 2021). Nonetheless, the interactive effects of the features of events and entity have rarely been investigated, even though they can reinforce each other and have a synergistic effect on creativity or innovation (Chen et al., 2021). Accordingly, this study is positioned to explore the impact of the synergistic effect of leader behavior and management events on employee creativity. In our research, we try to explain when AL has a further positive effect on employee creativity by revealing the interaction effect of appointment event criticality and discipline-focused AL.

Based on event system theory, event criticality is a significant characteristic that reflects “the degree to which an event is important, essential, or a priority” to an entity and typically triggers additional analyses and changes (Morgeson et al., 2015). We focus on “appointment event criticality” in this research. Appointment event refers to the experiences that an employee is designated for solving a new problem or undertaking a task as a team member (Zhao et al., 2021). In the workplace, each person may be appointed to get something done, such as a position change and starting a short-term project. These events provide contexts for employees and leaders to increase their understanding of each other. In previous qualitative studies, scholars found that discipline-focused AL could better motivate subordinates and reduce the promotion effect on work alienation by appointing (Zhao et al., 2021). We contend that appointment event criticality may augment the positive relationship between discipline-focus AL and employee creativity for two reasons. First, when employees accept an appointment deemed important for them from their leaders, they are more likely to understand the rigor of the leader because they view the appointment as specialized training rather than control of dominance. Here, they feel expected and trusted by their leaders. For example, if an employee concludes, “My direct leader expects me to complete a certain new task,” a logical attribution for the employee is “I must do well to live up to the leadership.” According to SDT, their needs for “competence” and “relatedness” are satisfied, thus they feel a strong sense of self-determination and want to complete the task better, which contributes to the improvement of creative self-efficacy.

Second, employees’ perceptions of leadership behaviors are influenced by the events experienced together. The more critical the appointment event is to the individuals, the stronger the individuals’ perception of leadership behavior (Zhao et al., 2021). In this study, criticality reflects the extent to which employees regard appointment events as “priority, essential, important” to them and typically triggers additional analyses and changes (Morgeson et al., 2015; Chen et al., 2021). Individuals with high perceived appointment event criticality are more confident because they think that discipline-focused authoritarian leaders communicate belief of “you can” by setting a high goal and maintaining high standards. As a result, individuals’ creative self-efficiency improves. Tierney and Farmer (2011) propose that perceived creativity expectations of direct leaders can promote employees’ assessment of the personal resources necessary for their creativity and help employees form their creativity efficacy beliefs. Conversely, individuals with low perceived appointment event criticality are less likely to feel the same conviction from their leader. Appointment event criticality regulates the degree of attention individuals need to pay to their leaders. Event system theory can explain the hypothesized interaction. We hypothesize the following:

*Hypothesis 3: Appointment event criticality moderates the positive relationship between discipline-focused AL and creative self-efficacy such that the relationship is stronger when appointment event criticality is higher.*

Thus far, we have hypothesized that creative self-efficacy mediates the relationship between discipline-focused AL and employee creativity, and that appointment event criticality moderates the relation between discipline-focused AL and creative self-efficacy. Integrating the arguments above, we propose a moderated mediation model. That is, appointment event criticality should strengthen the indirect impact of discipline-focused AL on employee creativity through creative self-efficacy. Taken together, our argument leads to the following hypothesis:

*Hypothesis 4: Appointment event criticality moderates the indirect effect of discipline-focused AL on employee creativity via creative self-efficacy, such that the indirect effect is stronger (i.e., more positive) when appointment event criticality is higher.*

**FIGURE 1:**
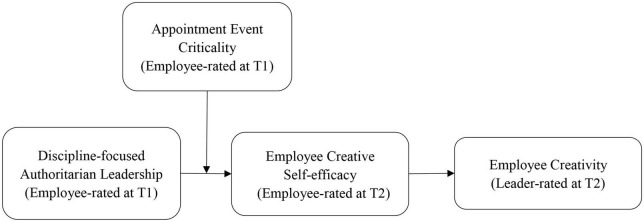
The hypothesized model.

The hypothesized model is shown in Figure 1.

## Materials and methods

### Procedure and samples

To test our hypotheses and avoid common method single source problems (Podsakoff et al., 2003), we carried out a two-wave data collection over 3 weeks, consistent with prior creativity studies (e.g., Huang et al., 2016). Prior studies demonstrated that the effect of antecedent variables (discipline-focused AL in our study) on employee outcomes (creative self-efficacy and employee creativity in our study) generally occurs after two weeks (cf. Wayne and Ferris, 1990; Chen et al., 2020). Thus, we consider a 3-week interval is appropriate for testing the hypotheses.

The data were collected from pairs of subordinates and their direct supervisors at seven medium-sized enterprises in Heilongjiang, Wuhan Provinces, and Beijing city, China. All these enterprises have a strong need for creative input from their employees to stay competitive in a rapidly changing market environment. These industries include advertising, media, software, and electronic engineering. First, we informed the general managers of the survey and sought their participation. In return, we would provide each enterprise with a data analysis report. Second, all employees were told that the study was just for the purposes of academics and would have no impact on individual performance. Third, we obtained a list of temporary personnel code numbers prepared by HR and coded each questionnaire accordingly to allow matching. HR then distributed each questionnaire to the corresponding employees without displaying their names. Instead, questionnaires distributed to the direct leaders would show the employee’s name. Fourth, participants in the study were given a special envelope with security features to seal the filled questionnaires. Finally, all questionnaires were returned to the research assistants to ensure they would remain anonymous.

In our first wave survey (Time 1), we administered questionnaires to 626 full-time employees to fill out the information on their demographics, discipline-focused AL and appointment event criticality. Three weeks after, the second wave survey (Time 2) began. We invited 529 employees whose questionnaires were usable at Time 1 to report their creative self-efficacy (response rate was 84.50%). Meanwhile, we invited the direct leaders of those 529 employees to rate each subordinate’s creativity. After removing 94 pairs due to invalid answers (e.g., all the items were given 1 or 5 points), missing data (e.g., the questionnaire was not fully answered), and failure of the verification test (e.g., “this is a verification item, and please select 2 points”), our final sample consisted of 435 matched pairs (48 leaders and 435 subordinates returned questionnaires, and every leader rated 9.06 subordinates; the valid response rate was 82.23%).

Among 435 subordinates, 36.6% were male and 63.4% were female. Their average age was 29.71 years (*SD* = 4.76), the average length of working years is 4.32 years (*SD* = 3.35), and the average tenure within the current organization was 1.18 years (*SD* = 0.53). In this sample, 3.9% graduated from high school, 31.3% held an associate degree, 59.3% held a bachelor’s degree, and the remaining 5.5% held a master’s degree or above.

### Measures

We used the translation and back translation method (Brislin, 1986). The Chinese version of the measurement was created (Brislin, 1986) based on the original English scale of employee creativity, creative self-efficacy and event criticality to ensure that semantic and concepts are equal. The Chinese version of the discipline-focused AL measures was used. All items are on a 5-point scale, ranging from 1 (“strongly disagree”) to 5 (“strongly agree”).

#### Discipline-focused authoritarian leadership

We measured discipline-focused AL using a 10-item scale from Chou and Cheng (2014). This scale was designed in the Chinese culture and used by Cheng et al. (2019, 2021). The scale’s Cronbach’s alpha was 0.84. All items are listed in Table 1.

**TABLE 1:** Discipline-focused authoritarian leadership scale.

Items
(1) My supervisor supervises the progress of work and asks me to do my best to achieve it.
(2) My supervisor asks me to strictly abide by the rules of task execution.
(3) My supervisor sticks to his work principles and does not allow me to violate them.
(4) My supervisor requires that my performance is not lower than the pre-set standard.
(5) My supervisor asks me to follow the core norms of the organization.
(6) My supervisor still asks me to improve my performance even when I have already reached my goals.
(7) My supervisor ask me to report to him immediately if there are any changes in my work.
(8) My supervisor fully realizes the details of my work.
(9) My supervisor controls the execution of my work.
(10) My supervisor will guide how I perform my work.

#### Creative self-efficacy

A three-item scale of creative self-efficacy developed by Tierney and Farmer (2002) was used. Sample items include “I am good at finding creative ways to solve problems” and “I have confidence in my ability to solve problems creatively.” The Cronbach’s alpha for this scale was 0.87.

#### Employee creativity

Supervisors evaluate the creativity of their direct reports according to the 13-item scale developed by Zhou and George (2001). Sample items include “He/she often has new and innovative ideas” and “He/she comes up with creative solutions to problems.” Cronbach’s alpha of the scale was 0.97.

#### Appointment event criticality

According to event system theory and relevant researches, the measurement of appointment event criticality was divided into a two-step procedure adopted by Morgeson (2005) and Morgeson and DeRue (2006). First, we defined the appointment event on the questionnaire and asked each employee to recall the experiences of being appointed by the direct supervisor and evaluate the experiences as a whole. The on-site research assistants were responsible for answering questions. Second, employees rated appointment event criticality using the three-item scale derived from Morgeson et al. (2015). Sample items include “These appointments are critical to the long-term success of my personal career” and “These appointments are important events in my work.” Cronbach’s alpha of the scale was 0.84.

#### Control variables

In line with prior creativity research, employees’ gender, age, education level and organization tenure was taken as control variables (cf., Gong et al., 2009; Gu et al., 2020), as these factors may affect the domain-relevant knowledge or expertise that is significant for creativity (Amabile, 1988; Chen et al., 2020). Another reason to control for demographic variables is that they tend to influence employee perceptions of leader’s behavior. In our research, we transformed the data for two control variables (i.e., employees’ gender, education level), which were originally categorical variables. Specifically, male = 1, female = 2; high school and below = 1, college = 2, undergraduate = 3, master = 4, doctor = 5.

## Results

### Preliminary analyses

To provide evidence that our measured constructs were distinguishable, before hypothesis testing, we performed a set of confirmatory factor analysis (CFA) to assess the discriminant validity of discipline-focused Al, employee creative self-efficacy, employee creativity, and appointment event criticality (cf., Gadeyne et al., 2018; Kim et al., 2021; Lian et al., 2022). The results are shown in Table 2.

**TABLE 2:** Results of confirmatory factor analysis.

	Variables	χ^2^	df	Δχ^2^/Δ df	RMSEA	SRMR	CFI
Four factors	DAL, AEC, CSE, EC	1036.58	371	—	0.06	0.04	0.91
Three factors	DAL, AEC, CSE + EC	1681.34	374	644.76/3	0.09	0.07	0.83
Two factors	DAL + AEC, CSE + EC	2096.09	376	1059.51/5	0.10	0.08	0.78
One factor	DAL + AEC + CSE + EC	3373.91	377	2337.33/6	0.14	0.15	0.61

DAL, discipline-focused authoritarian leadership; AEC, appointment event criticality; CSE, creative self-efficacy; EC, employee creativity; RMSEA, root mean square error of approximation; SRMR, standardized root mean square residual; CFI, comparative fit index.

As exhibited in Table 2, the four-factor model had the best fit [χ^2^(371) = 1036.58, RMSEA = 0.06, SRMR = 0.04, CFI = 0.91] over the three-factor model, in which creative self-efficacy and employee creativity were combined [Δχ^2^(3) = 633.76, *p* < 0.01], and better than the alternative models. Therefore, the research reserved the four-factor model (cf. Gadeyne et al., 2018; Kim et al., 2021).

Additionally, a Harman one-factor test was also performed in this study. When not rotated, the first common factor accounts for 30.37% of the total loading, which is less than 40%, indicating that there is no common method bias in this study, and subsequent research can be carried out.

Table 3 provides the descriptive statistics, reliability and correlation of variables. Then, based on the methods suggested by Podsakoff et al. (2003), we tested the effectiveness of incorporating a potential common method factor into the four-factor model. The general method factor accounts for only 3.3% of the total deviation of the model interpretation, which is lower than the average variance (Williams et al., 1989), which shows that common method variance was not a pervasive problem.

**TABLE 3:** Descriptive statistics, reliabilities, and correlations^a^.

Variables	Mean	*SD*	1	2	3	4	5	6	7	8
(1) Gender^b^	1.63	0.48								
(2) Education^c^	2.66	0.64	–0.11*							
(3) Age	29.71	4.76	0.00	–0.04						
(4) Tenure	4.32	3.35	–0.06	–0.02	0.54**					
(5) DAL	3.71	0.65	–0.10*	–0.08	0.18**	0.17**	(0.84)			
(6) AEC	4.06	0.81	–0.01	–0.04	0.10*	0.06	0.32**	(0.84)		
(7) CSE	3.79	0.75	–0.13**	0.02	0.00	0.01	0.11*	0.11*	(0.87)	
(8) EC	3.80	0.81	–0.08	0.06	–0.02	0.07	–0.02	–0.03	0.10*	(0.96)

^a^N = 435. ^b^1 = male, 2 = female. ^c^1 = high school and below, 2 = college, 3 = undergraduate, 4 = master, 5 = doctor. DAL, discipline-focused authoritarian leadership; AEC, appointment event criticality; CSE, creative self-efficacy; EC, employee creativity. *p < 0.05, **p < 0.01, ***p < 0.001.

### Hypotheses testing

This study conducted a series of hierarchical regression analyzes using SPSS to check our hypothesis To test the indirect and conditional indirect effects, we used PROCESS (Hayes, 2013) and constructed 95% bias-corrected confidence intervals using 20,000 bootstrap samples. Compared with percentile bootstrap confidence intervals, this method avoids more errors (Preacher and Hayes, 2008).

The results of the hierarchical regression analysis are reported in Table 4. As shown in Model 1a, there is a positive correlation between discipline-focused Al and creative self-efficacy (*b* = 0.12, *p* < 0.05), thereby supporting Hypothesis 1. Model 2b shows that there is a positive relationship between creative self-efficacy and employee creativity (*b* = 0.10, *p* < 0.05). Moreover, the 95% confidence intervals of the indirect effect of discipline-focused Al on employee creativity via creative self-efficacy excluded zero (estimate = 0.014, 95%CI [0.001, 0.041]). Accordingly, Hypothesis 2 is supported.

**TABLE 4:** Regression results (coefficients and standard errors)^a^.

Variables	Outcome: CSE (T2)			Outcome: EC (T2)	
		
	Model 1a	Model 1b	Model 1c	Model 2a	Model 2b
Constant	3.71*** (0.38)	3.52*** (0.39)	3.51*** (0.39)	4.21*** (0.41)	3.83*** (0.45)
Gender^b^	–0.19* (0.08)	–0.19* (0.08)	–0.19* (0.08)	–0.12 (0.08)	–0.10 (0.08)
Education^c^	0.01 (0.06)	0.02 (0.06)	0.02 (0.06)	0.07 (0.06)	0.06 (0.06)
Age	–0.00 (0.01)	–0.00 (0.01)	–0.01 (0.01)	–0.01 (0.01)	–0.01 (0.01)
Tenure	–0.00 (0.01)	–0.00 (0.01)	0.00 (0.01)	0.03 (0.01)	0.03 (0.01)
DAL (T1)	0.12* (0.06)	0.08 (0.06)	0.07 (0.06)	–0.04 (0.06)	–0.05 (0.06)
AEC (T1)		0.08 (0.05)	0.10* (0.05)		
DAL × AEC			0.15* (0.07)		
CSE (T2)					0.10* (0.05)
Δ*R*^2^		0.01	0.01*		0.01*

^a^N = 435. ^b^1 = male, 2 = female. ^c^1 = high school and below, 2 = college, 3 = undergraduate, 4 = master, 5 = doctor. DAL, discipline-focused authoritarian leadership; AEC, appointment event criticality; CSE, creative self-efficacy; EC, employee creativity. *p < 0.05, **p < 0.01, ***p < 0.001.

**FIGURE 2:**
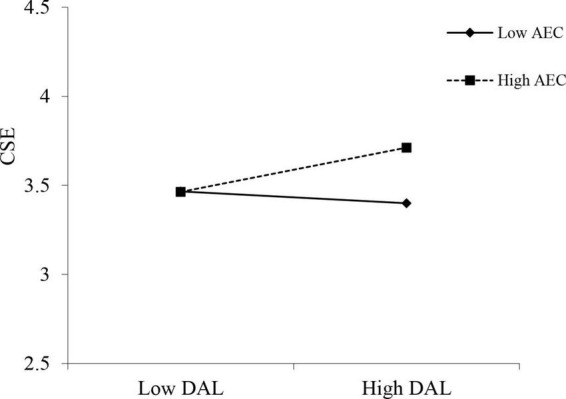
Interaction effect of discipline-focused authoritarian leadership and the criticality of appointment event on creative self-efficacy. DAL, discipline-focused authoritarian leadership; AEC, appointment event criticality; CSE, creative self-efficacy.

Model 1c test Hypothesis 3. The interaction between discipline-focused Al and appointment event criticality was significant (*b* = 0.15, *p* < 0.05). The simple slope test shows that when the criticality of appointment events is low (–1 SD), the effect of discipline-focused Al is insignificant (*b* = –0.05, n.s.), whereas it became significant and positive (*b* = 0.19, *p* < 0.01) with higher levels of appointment event criticality (+1 SD). Figure 2 plots the interaction. Hypothesis 3 is empirically supported.

To test Hypothesis 4, we estimated the 95% confidence intervals (CIs) to justify the significance of conditional indirect effects. With higher levels of appointment event criticality (+1 SD), the indirect effect of discipline-focused Al on employee creativity via creative self-efficacy (estimate = 0.023, 95%CI [0.003, 0.063]) was positive and significant. When appointment event criticality was lower (–1 SD), the indirect effect became non-significant (estimate = –0.005, 95%CI [–0.033, 0.011]). Therefore, Hypothesis 4 received support.

## Discussion

Given the fact that leaders sometimes engage in authoritarian behavior with the purpose of individual and organizational improvement (Guo et al., 2018; Zhao et al., 2022), this study investigated the role of discipline-focused AL in employee creativity and challenged the dominant assumption of AL in two ways. First, the previous literature excessively emphasized the dark side of AL but ignored its bright side (Chou et al., 2010; Chou and Cheng, 2014). As a result, the academic research on AL is one-sided, leading to inconsistent results of AL on employee creativity, which have not been explained clearly. In this study, we turned our focus to the positive aspects and raised the possibility that AL promotes employee creativity. Consistent with our findings, Gu et al. (2020) and Zhang et al. (2021) came to a similar conclusion.

Furthermore, we elicited the concept of appointment event criticality and proposed the positive moderating effect of appointment event criticality on the relationship between discipline-focused AL and employee creativity, which offers insights into workplace events and entity (i.e., individual, team, organization) variables. Our findings verified the “integration theory-building approach” in the event system theory (Morgeson et al., 2015). In the event study, we can consider the characteristics of the entity and the events experienced by the entity simultaneously, and study the synergistic effect on the outcome variables. In this respect, our findings are consistent with the conclusions of previous studies (e.g., Chen et al., 2020; Liu et al., 2021; Zhao et al., 2022).

To sum up, our study provides a better understanding of both AL and employee creativity with theoretical and practical significance.

### Theoretical implications

First, our study enriches the current literature on the types of AL by conceptualizing discipline-focused AL. Discipline-focused AL can be viewed as a “stressful challenge” (Cheng et al., 2021), presenting a more active leadership style that motivates employees to seek higher performance. Some studies indicate that the strict style, high performance expectations, and high standards would stimulate employees’ “competence” motivation at work and employees’ belief that self-worth and self-realization could be gained (e.g., Zhao et al., 2021). However, few researchers have theoretically developed discipline-focused AL, even though some scholars have argued that it could bring intrinsic motivation for individuals to pursue higher performance (Chou and Cheng, 2014; Cheng et al., 2021). Our study contrasts with the work of Wang et al. (2022), in which AL as a comprehensive construct negatively affects creativity through creative self-efficacy. We view AL as a two-dimensional construct containing positive behaviors and negative behavior (Chou and Cheng, 2014) and examine its impact on employee creativity from its bright side. Our research expands the theory of AL and lays a foundation for future study on its “light side.”

Second, our study deepens the understanding of the relationships between control behavior of leaders and employee creativity, especially in the context of Chinese culture, although creativity researchers are likely to accept the views that AL negatively correlates with creativity (Zhang et al., 2011). In our research, discipline-focused AL emphasizes the control of standards, not damaging the personal dignity of employees, so this control is more acceptable to employees (Wang and Xing, 2019). The results of our study echo the findings of Zhang et al. (2021), who demonstrated that AL has a positive effect on employee innovation behavior in Chinese culture. Previous empirical studies have shown that employee innovation behavior, creative self-efficacy, and creativity are highly correlated (Tierney and Farmer, 2004). It is easier for people influenced by Chinese culture to understand the control of AL (Zhang et al., 2021), such as focusing on hierarchical systems and having considerable executive power demands (Wang and Guan, 2018). Our research may provide theoretical researchers with additional perspectives on when and how AL affects employee creativity in the context of Eastern organizational cultures.

Third, our research extends SDT and event system theory (Morgeson et al., 2015) to prove that leader behaviors and event characteristics may interact and have synergetic effects on employees’ cognition and behavior. We expand an essential aspect of situational factors, which moderate the relationship between leadership and employee creativity. Although previous studies have broadened our understanding of the boundary conditions under which leadership influences employee creativity, they focus solely on the static features of contexts and individuals, which is not sufficient to explain the antecedents of employee creativity in today’s increasingly dynamic workplace (Chen et al., 2020). In this study, we introduce appointment event criticality based on the perspective of appointment, which leaders and employees often experience but tend to overlook. Our results show that discipline-focused AL has an indirect impact on employee creativity through creative self-efficiency, which is stronger when appointment event criticality is higher. Our study shows that research should pay more attention to discrete and acute events, such as appointment events, which may have a significant impact on individuals in the organization.

### Practical implications

This study provides a new view of management practice for AL. Different from previous studies, our findings imply that not all AL behavior will damage employee self-worth and creativity. Previous research results indicate that AL can indeed have negative effects on employees (e.g., Chan et al., 2013; Zhang and Xie, 2017). However, when a directive leadership approach is necessary, authoritarian leaders can better play their strengths and avoid their weaknesses. Authoritarian leaders should demonstrate stressful challenging behavior by demanding full effort from subordinates and setting high-level goals for them. These leadership behaviors of AL can motivate subordinates’ creative self-efficiency, and increase their creativity. Just as a Chinese proverb says, “capable are pupils trained by strict masters.”

More importantly, our study shows that appointment event criticality facilitates a positive indirect impact of discipline-focused AL on employee creativity. In this study, the synergistic of discipline-focused AL and appointment event criticality provides clear guidance to leaders and managers on when, whether, and how to motivate subordinates’ creative self-efficiency, which leads to creative work. Consequently, managers can enhance subordinates’ positive perception of leaders through important appointment events when conducting management behavior. Event system theory argues that events are part of the context or situation, emphasizing that entities can actively create events and arouse the contextual perception of employees, which in turn change employees’ cognitions and behaviors (Liu and Liu, 2017). Accordingly, employees can take the initiative to seek important tasks from managers. Current studies have shown that the appointment event criticality can help leaders establish positive images among employees and enhance employees’ positive perceptions of leaders (Zhao et al., 2021). Therefore, organizations should not only focus on the stable features of individuals and organizations, such as leadership style, organizational identity, and organizational support, but also focus on the appointment events that can significantly affect employees. Managers may establish an appointment mechanism that allows each employee to undertake major tasks, and let the positive effects of appointment events empower employees. These appointment events can stimulate positive cognition to the leader, and are conducive to employees due to acquiring a wider range of skills, which is advantageous in future promotions.

### Limitations and future directions

Despite the theoretical and practical contributions, our research has limitations in the following aspects. First, employee creativity was evaluated by supervisors. Whether an employee is creative or not does not depend entirely on the evaluation of the leader. Although measuring employee creativity from the perspective of leaders has been adopted by many studies to address common method bias and social desirability, we remain concerned that the evaluation of employee creativity was not objective enough. Therefore, future research seeks to rate employ creativity in multiple ways.

Second, our study explains the relationship between discipline-focused AL and employee creativity from the perspective of motivation and basic psychological needs. There is a chance that a similar motive or cognitive variable exists in the relationship between discipline-focused AL and employee creativity, which could be elicited in future studies.

Third, we focused on appointment events, the collection of micro events elicited by leaders, and investigated its criticality’s synergistic effect with discipline-focused AL. As Morgeson et al. (2015) and Chen et al. (2021) suggested, endogenous events (i.e., events created by oneself) and macro events also require attention. We believe that many types of events play a regulating role in the leadership effect on employee creativity, and scholars can conduct further research in this regard.

## Data availability statement

The raw data supporting the conclusions of this article will be made available by the authors, without undue reservation.

## Author contributions

HZ and QS contributed to conception and design of the study. ML performed the statistical analysis. HZ wrote the first draft of the manuscript. CH edited and polished the second draft. LZ was responsible for the entire data acquisition process. All authors contributed to manuscript revision, read, and approved the submitted version.
